# Arsenic Primes Human Bone Marrow CD34+ Cells for Erythroid Differentiation

**DOI:** 10.1155/2015/751013

**Published:** 2015-06-11

**Authors:** Yuanyuan Zhang, Shasha Wang, Chunyan Chen, Xiao Wu, Qunye Zhang, Fan Jiang

**Affiliations:** ^1^Key Laboratory of Cardiovascular Remodeling and Function Research, Qilu Hospital, Shandong University, Jinan, Shandong 250012, China; ^2^Department of Oncology and Pathology (CCK), R8:03, Karolinska University Hospital, Solna, 17176 Stockholm, Sweden; ^3^Department of Hematology, Qilu Hospital, Shandong University, Jinan, Shandong 250012, China

## Abstract

Arsenic trioxide exhibits therapeutic effects on certain blood malignancies, at least partly by modulating cell differentiation. Previous *in vitro* studies in human hematopoietic progenitor cells have suggested that arsenic may inhibit erythroid differentiation. However, these effects were all observed in the presence of arsenic compounds, while the concomitant cytostatic and cytotoxic actions of arsenic might mask a prodifferentiating activity. To eliminate the potential impacts of the cytostatic and cytotoxic actions of arsenic, we adopted a novel protocol by pretreating human bone marrow CD34+ cells with a low, noncytotoxic concentration of arsenic trioxide, followed by assaying the colony forming activities in the absence of the arsenic compound. Bone marrow specimens were obtained from chronic myeloid leukemia patients who achieved complete cytogenetic remission. CD34+ cells were isolated by magnetic-activated cell sorting. We discovered that arsenic trioxide enhanced the erythroid colony forming activity, which was accompanied by a decrease in the granulomonocytic differentiation function. Moreover, in erythroleukemic K562 cells, we showed that arsenic trioxide inhibited erythrocyte maturation, suggesting that arsenic might have biphasic effects on erythropoiesis. In conclusion, our data provided the first evidence showing that arsenic trioxide could prime human hematopoietic progenitor cells for enhanced erythroid differentiation.

## 1. Introduction

Inorganic arsenic, mainly in the form of arsenic trioxide (ATO), has been proved to be effective in the treatment of certain blood malignancies [1, 2]. The pharmacological effects of ATO are mainly through modulation of the posttranslational modification and proteasomal degradation of key proteins involved in the pathogenesis of these diseases [1, 2]. In addition, induction of differentiation and apoptosis of leukemic cells may also have an essential role in the therapeutic action of ATO [3]. On the other hand, arsenic is also a major environmental toxin, and exposure to arsenic in humans may have proinflammatory and carcinogenic effects in multiple organs, including the hematopoietic system [4]. Previous* in vitro* studies in human hematopoietic progenitor cells have suggested that arsenic may inhibit erythroid differentiation [5, 6]. However, these effects were all observed in the presence of arsenic throughout the process of differentiation induction. Since ATO has cytostatic and cytotoxic actions in human hematopoietic cells [5, 6], these effects may potentially influence the results of the subsequent differentiation assay.

To eliminate possible impacts of the disturbed cell proliferation on the readout of differentiation functions, we adopted a novel protocol by pretreating human bone marrow (BM) CD34+ cells with a low, noncytotoxic concentration of ATO, followed by assaying the colony forming activities in the absence of ATO. Interestingly, we discovered an unexpected priming effect of ATO for enhanced erythroid colony forming activity, which was accompanied by a decrease in the granulomonocytic differentiation function.

## 2. Methods

### 2.1. Collection and Purification of BM Cells

This study was approved by the Human Ethics Committee of Qilu Hospital. Written consent forms were obtained from all participants before sample collection. BM specimens were obtained from chronic myeloid leukemia patients during routine follow-up tests. All patients had received imatinib treatment and achieved complete cytogenetic remission. Surplus BM samples were spared after the clinical test and total mononuclear cells were isolated by gradient density centrifugation using Ficoll-Paque. Cells were suspended in cryopreservation media containing 65% RPMI 1640, 25% normal human serum, and 10% DMSO and temporarily stored in liquid nitrogen. For CD34+ cell purification, cells from different patients were thawed and pooled together to ensure enough cell number for experimentation. CD34+ cells were purified by magnetic-activated cell sorting using a human CD34 MicroBead Kit (Miltenyi). This procedure also eliminated most of the dead cells. This approach has been widely used in previous studies to purify human CD34+ progenitors [7–9]. The purity of the isolated cells for CD34 expression was >90% as determined by flow cytometry. The resultant CD34+ cell preparation was also examined with fluorescent in situ hybridization (using a kit from Cytocell, Cambridge, UK), and Ph+ cells could not be detected.

### 2.2. Cell Culture, Treatment, and Colony Forming Unit (CFU) Assay

CD34+ cells were cultured in the StemPro-34 serum-free medium system (Life Technologies) supplemented with 2 mM glutamine, 100 *μ*M 2-mercaptoethanol, in the absence or presence of the following cytokines (concentration in ng/mL): Flt3-ligand 100, stem cell factor 100, interleukin-3 20, and granulocyte-colony stimulating factor 20 (all from Life Technologies), as described previously [10] and recommended in the manufacturer's protocol. ATO (a gift from Dr. Zhu Chen's Laboratory, Shanghai Institute of Hematology) was dissolved in PBS and added into the culture for 7 days. For CFU assay, cells were washed off ATO and colony forming cells were detected using the MethoCult system from Stemcell Technologies (#H4434) according to the manufacturer's directions. This method has been validated by other groups for studying human hematopoietic progenitor cell differentiation [11, 12]. Colonies were counted on day 16. Erythroleukemic K562 cells were cultured in RPMI 1640 medium supplemented with 10% fetal calf serum.

### 2.3. Flow Cytometry Analysis

Cell viability was assessed by detecting necrotic and apoptotic cells within the population with flow cytometry (FACSCalibur system (BD Biosciences, Mountain View, CA, USA)), using an Annexin V-FITC Apoptosis Detection Kit (BD #556547), which can distinguish necrotic, early apoptotic, and late apoptotic cells. Cell surface expressions of glycophorin A and CD41a were also measured by flow cytometry using FITC-conjugated monoclonal anti-human CD41a (BD #557296) and anti-human glycophorin A (R&D #FAB12281P) antibodies.

### 2.4. Quantitative Real-Time PCR (qPCR)

Expressions of human alpha, beta, and gamma hemoglobin genes (HBA, HBB, and HBG) in K562 cells were measured by qPCR using the SYBR green method (SsoFast EvaGreen Supermix from Bio-Rad, Hercules, CA, USA) as described previously [13]. The primer sequences were from previous reports [14, 15].

### 2.5. Western Blot

Western blot was performed as described using a monoclonal anti-human hemoglobin-zeta antibody (R&D #MAB7708) [13].

### 2.6. Data Analysis

Data were expressed as mean ± SEM. Unpaired two-sided *t*-test was used for statistical analysis. A *P* value of < 0.05 was considered as statistically significant.

## 3. Results

**Figure 1:**
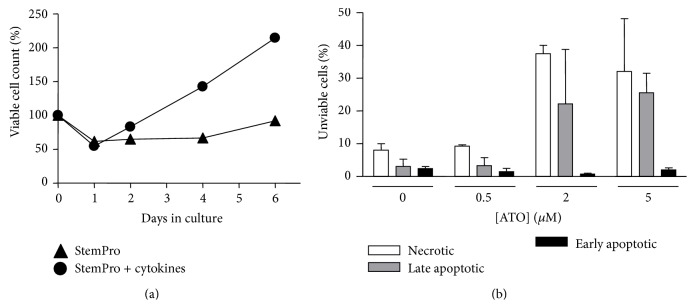
Proliferation and ATO-induced cytotoxicity of BM CD34+ cells. (a) Comparison of cell proliferation when cultured in StemPro-34 medium without and with added cytokines. Viable cells were identified by trypan blue exclusion. Data are mean from two experiments. (b) Effects of ATO at different concentrations on cell viability assessed by flow cytometry. Cells were treated with ATO for 7 days in StemPro-34 (*n* = 2).

**Figure 2:**
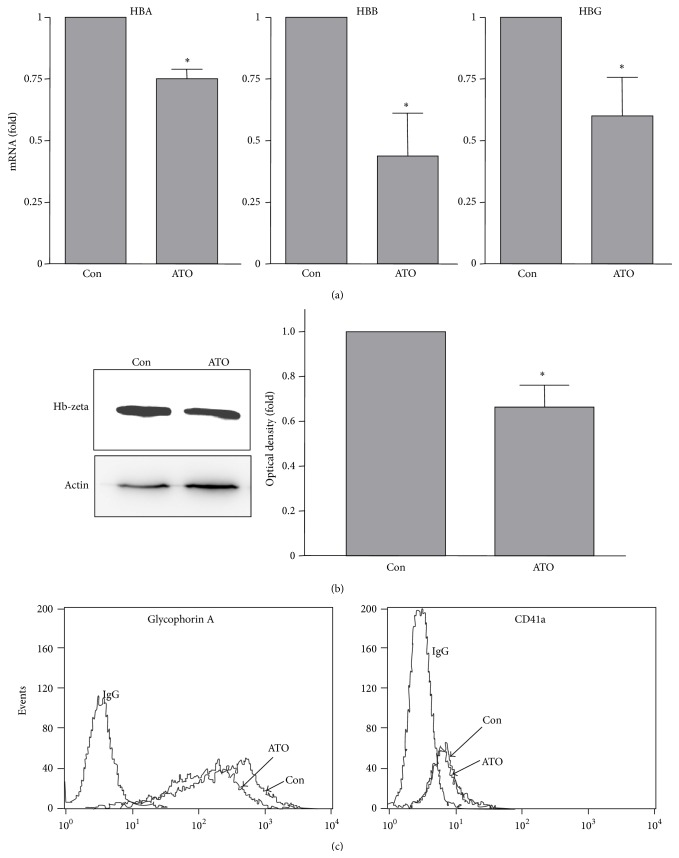
Effects of 0.5 *μ*M of ATO (treatment for 5 days) on (a) mRNA expression levels of hemoglobin genes (qPCR), (b) protein expression of hemoglobin-zeta (western blotting), and (c) cell surface expression levels of glycophorin A and CD41a (flow cytometry) in K562 cells. Data were mean ± SEM. ^*∗*^ *P* < 0.05 versus control, unpaired *t*-test, *n* = 3-4.

We first optimized the culture condition for the progenitor cells. We found that elimination of the supplemented cytokines as reported in the StemPro-34 medium rendered the cells nonproliferating while it maintained a good viability (Figure 1(a)). Under this culture condition, we next screened the effects of ATO of different concentrations on CD34+ BM cell viability. Following a 7-day treatment period, ATO at 0.5 *μ*M showed no effects on cell apoptosis or necrosis, while ATO at 2 and 5 *μ*M increased the proportion of cells in the necrotic and late apoptotic status by 3-4-fold (Figure 1(b)). To elucidate the effects of ATO on differentiation of the progenitor cells, we pretreated the cells with ATO at 0.5 *μ*M for 7 days and then performed CFU assay in the absence of ATO. We found that there was a significant increase in the proportion of erythroid colonies in ATO-pretreated cells, while the total CFU activity of the progenitor cells was not changed (see Table 1). The proportion of granulocyte/macrophage colonies was reduced in contrast. To test the effects of ATO at the same concentration on erythrocyte maturation, we treated K562 cells for 5 days and measured the expression levels of hemoglobin with qPCR. We found, however, ATO significantly decreased the expression levels of HBA, HBB, and HBG (Figure 2(a)). Using western blot, we also showed that ATO significantly reduced the expression level of hemoglobin protein (Figure 2(b)). Moreover, we found that ATO significantly reduced the expression level of glycophorin A (marker of erythrocyte) in K562 cells but had no effect on the level of CD41a (marker of megakaryocyte) (Figure 2(c)).

## 4. Discussion

**Figure 3:**
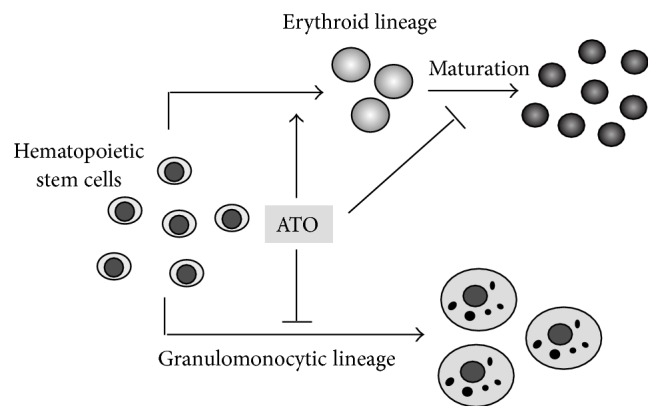
A diagram summarizing the major findings of this study. We provided the first evidence showing that ATO could prime human hematopoietic progenitor cells for an enhanced erythroid differentiation, at the expense of decreased granulomonocytic differentiation.

The major finding of the present study is that ATO, at a noncytotoxic concentration, primes human hematopoietic progenitor cells for erythroid differentiation, which is accompanied by decreased granulomonocytic differentiation (summarized in Figure 3). This phenomenon has not been reported by other studies. Arsenic intake has been found to cause megaloblastic anemia [16, 17]. However, it was also observed that arsenic intoxication increased human BM cellularity and erythroid hyperplasia [17]. It is not clear whether the erythroid hyperplasia was a direct effect of arsenic on hematopoietic stem cells or a compensatory response following anemia. Our data may provide an explanation to these results, indicating that arsenic may directly regulate differentiation of human hematopoietic progenitors.

Increased oxidative stress is thought to have a pivotal role in mediating the biological and toxicological effects of arsenic [18]. Notably, several studies have demonstrated that increased reactive oxygen species may reduce the stemness of hematopoietic stem cells [19, 20] and promote lineage commitment [21]. In a recent study, it was demonstrated that intracellular reactive oxygen species might be involved in promoting erythroid lineage differentiation of human cord blood CD34+ cells under mild hyperthermia stress condition [22]. In light of these findings, we propose that the observed erythroid expansion induced by ATO may be mediated by a redox-dependent mechanism that enhances differentiation to the erythroid lineage. Supporting this notion, a very recent study showed that arsenic disulfide could trigger erythroid differentiation in myeloid leukemic cell lines [23].

Although ATO promoted expansion of erythroid colonies in human primary BM cells, we demonstrated that, in K562 cells, ATO decreased the expression of hemoglobin genes and decreased the level of the erythrocyte marker glycophorin A. This finding is consistent with that reported by previous studies [5], suggesting that ATO inhibits erythrocyte maturation. Taken together, our data suggest that ATO may have a biphasic effect on erythropoiesis. On the other hand, it has been shown that ATO increases the expression of the megakaryocyte marker CD41a in the human erythroleukemic cell line HEL [5]. However, under our experimental conditions, we did not find any effect of ATO on CD41a expression in K562 cells. This discrepancy may indicate that the responsiveness of these cells to ATO is different.

Human leukemic diseases are thought to be clonal pathologies initiated at the level of either pluripotent hematopoietic stem cells or early multipotent progenitors [24], and blockade of differentiation of the leukemic cells at a distinct stage in cellular maturation is a characteristic feature of leukemia [25]. Differentiation therapy is an effective treatment strategy for certain types of leukemia such as acute promyelocytic leukemia [25, 26]. Indeed, acute promyelocytic leukemia is also sensitive to arsenic trioxide, while the mechanisms of the beneficial effects of arsenic are still not entirely understood [26, 27]. Our results raise a possibility that arsenic-induced differentiation may also be explored for novel combinatorial therapy strategies. An outstanding question is whether arsenic also affects differentiation of leukemic stem cells or progenitor cells and importantly whether arsenic-induced differentiation alters the sensitivity of leukemic stem/progenitor cells to other chemotherapeutic agents.

## 5. Conclusions

In summary, our data provided the first evidence indicating that ATO could prime human hematopoietic progenitor cells for an enhanced erythroid differentiation. The physiological or pathophysiological significance of ATO-induced regulation of hematopoietic differentiation, however, remains to be elucidated.

## Figures and Tables

**Table 1:** Effects of ATO pretreatment on colony forming activities of human BM CD34+ cells.

	Control	ATO	*P*
Total CFU (per 10^3^ cells)	58.0 ± 5.7	69.5 ± 5.6	0.2
CFU-E (%)	4.1 ± 0.9	14.9 ± 3.8	0.03
BFU-E (%)	34.4 ± 2.5	48.9 ± 3.7	0.02
Total E (%)	38.5 ± 2.3	63.3 ± 5.6	0.007
CFU-GM (%)	59.7 ± 2.3	33.3 ± 5.6	0.005
CFU-GEMM (%)	1.8 ± 0.2	3.3 ± 0.5	0.03

Bone marrow CD34+ cells primed with 0.5 *μ*M were seeded in 6-well plates (1000 cells per well) and colonies counted on day 16. CFU: colony forming unit; BFU: burst forming unit; E: erythroid; GM: granulocyte macrophage; GEMM: granulocyte, erythroid, macrophage, and megakaryocyte. Unpaired *t*-test was performed for comparison of the mean data (mean ± SEM, experiments in quadruplicate).
